# Knowledge and self-reported practice of laboratory biosafety among medical laboratory professionals in selected public and private hospitals in Mogadishu, Somalia: a cross-sectional study

**DOI:** 10.3389/fpubh.2026.1916020

**Published:** 2026-10-28

**Authors:** Omar Sidow Zubair, Ahmed Abdi Yonis, Bashir Abukar Mohamud, Mohamed Hassan Mohamud

**Affiliations:** 1 Faculty of Postgraduate Studies, Hormuud University, Mogadishu, Somalia; 2 Department of Medical Microbiology and Immunology, Faculty of Health Sciences, University of Nairobi, Nairobi, Kenya

**Keywords:** knowledge and self-reported practice, laboratory biosafety, medical laboratory professionals, Mogadishu hospitals, Somalia

## Abstract

**Background:**

Laboratory biosafety protects healthcare workers, patients and the environment, yet evidence from fragile and resource-limited settings remains scarce. In Somalia, previous work has assessed infection prevention and control at the facility level, but the biosafety knowledge and practice of laboratory professionals themselves, and the relationship between the two, have not been characterized. This study assessed knowledge and self-reported practice of laboratory biosafety, and factors associated with good practice, among medical laboratory professionals in public and private hospitals in Mogadishu, Somalia.

**Methods:**

A facility-based cross-sectional study was conducted among 164 eligible medical laboratory professionals in 12 hospitals selected by stratified random sampling from the public and private hospital sectors in Mogadishu. All eligible professionals within the selected hospitals were enumerated; the study is therefore a complete enumeration within sampled facilities rather than a census of all laboratory professionals in Mogadishu. Data were collected with a structured, self-administered questionnaire adapted from the WHO Laboratory Biosafety Manual and previously validated knowledge–practice instruments. Knowledge was measured with 8 dichotomously scored items (range 0–8; KR-20 = 0.60) and self-reported practice with 9 dichotomous items (range 0–9; KR-20 = 0.52), both classified using pre-specified modified Bloom’s cut-off points. Distributions were assessed with the Shapiro–Wilk test. Associations were examined using the Pearson chi-square test and Spearman’s rank correlation, and factors associated with good practice using univariable and multivariable binary logistic regression.

**Results:**

The median knowledge score was 7 (IQR 5–7) of 8 and the median practice score 9 (IQR 8–9) of 9; both departed significantly from normality. Knowledge was high for hand hygiene (89.0%) and personal protective equipment (86.6%) but low for infectious waste colour-coding (53.7%). Overall, 83 participants (50.6%) had good knowledge, 51 (31.1%) moderate and 30 (18.3%) poor. Self-reported practice was high for lab coat use (98.2%), hand hygiene (98.2%) and glove use (97.6%), and lower for equipment inspection (78.7%) and accident reporting (81.7%); 134 participants (81.7%) were classified as having good practice. A statistically significant positive association was observed between knowledge level and practice level (*χ*^2^ = 19.58, df = 2, *p* < 0.001; Cramer’s *V* = 0.35; Spearman’s *ρ* = 0.296, *p* < 0.001), with good practice reported by 94.0, 74.5 and 60.0% of those with good, moderate and poor knowledge, respectively. In multivariable analysis, each additional correct knowledge answer independently increased the odds of good practice (aOR 1.73, 95% CI 1.33–2.24, *p* < 0.001), as did participation in a safety drill in the preceding year (aOR 2.80, 95% CI 1.09–7.15, *p* = 0.032). Only 64.6% of participants had taken part in such a drill.

**Conclusion:**

Medical laboratory professionals in Mogadishu hospitals demonstrated moderate biosafety knowledge and high self-reported biosafety practice, although there were gaps in knowledge of infectious waste colour-coding. Knowledge scores were positively associated with self-reported practice, while recent participation in a safety drill was independently associated with reported participants’ practice. These findings suggest that regular safety drills and targeted reinforcement of infectious waste-management procedures may be useful areas for strengthening biosafety practices. However, the high reported practice scores and their potential effect underscore the need for future studies to assess biosafety practices through direct observation.

## Introduction

Biosafety comprises the principles, technologies and practices implemented to prevent unintentional exposure to pathogens and toxins or their accidental release. International guidance such as the World Health Organization (WHO) Laboratory Biosafety Manual, 4th edition (LBM4) promotes a risk-based approach emphasising risk assessment, a hierarchy of controls, continuous training and locally appropriate safety management systems (1). This represents a deliberate shift away from rigid biosafety-level classification toward a flexible framework that tailors safety measures to the specific risks posed by a given procedure, pathogen and local context, recognising that a uniform approach is neither feasible nor effective across the diverse range of laboratory settings worldwide (2). Terminological distinction is important at the outset, because the two terms are frequently used interchangeably. Laboratory safety is the broader construct, encompassing chemical, physical, electrical, fire, radiological and ergonomic hazards in addition to biological ones. Laboratory biosafety refers specifically to the containment principles, technologies and practices applied to prevent unintentional exposure to, or accidental release of, biological agents (1). The instrument used here assesses containment-related knowledge and behaviors personal protective equipment, specimen handling, biosafety cabinet use, infectious waste segregation, hand hygiene and accident reporting—with a single item on chemical spill response included because chemical spills are a routine co-hazard during biological specimen processing (3). The construct assessed is therefore laboratory biosafety, and this term is used throughout.

Clinical laboratories handle diverse pathogens and hazardous materials, placing laboratory professionals at increased risk of occupational exposure, laboratory-acquired infections and accidental injuries. Globally, an estimated 500,000 laboratory workers are potentially exposed to infectious agents annually, with documented risks associated with improper use of personal protective equipment (PPE), inadequate biosafety training and poor adherence to standard operating procedures (4). Common exposure routes include needlestick and sharps injuries, mucocutaneous splashes, inhalation of infectious aerosols generated during centrifugation and pipetting, and ingestion through poor hand hygiene; each is in principle preventable through consistent adherence to established biosafety practices (5).

Despite well-established international guidelines, translating biosafety knowledge into consistent everyday practice remains a persistent challenge even in well-resourced health systems. A growing occupational health literature indicates that knowledge of correct procedures does not by itself guarantee compliance, because practice is shaped by an interplay of individual, organisational and systemic factors including workload pressure, availability of protective equipment and consumables, supervisory oversight, institutional safety culture and the perceived consequences of non-compliance (5). Comparable dissociations between knowledge and behavior have been reported for nursing personnel applying standard and transmission-based precautions (6), and for health workforces assessed for outbreak readiness, where high awareness has coexisted with low operational preparedness (7). This is precisely why knowledge and practice must be measured as separate constructs within the same population rather than one being treated as a proxy for the other: measuring knowledge alone would risk misstating the safety of the workforce, while measuring practice alone would leave open whether any observed deficit is remediable by training or requires structural intervention. Establishing the relationship between the two within a single sample is what distinguishes these two policy responses.

Low- and middle-income countries face compounding challenges in maintaining laboratory biosafety, including constrained financial resources, irregular training opportunities, ageing infrastructure, inconsistent supply chains for PPE and safety consumables, and weak regulatory enforcement (8). In many African countries biosafety programmes remain fragmented and poorly standardised, and laboratory workers frequently report insufficient access to PPE, irregular in-service training and limited institutional support (9). These deficiencies are particularly consequential given recurrent infectious disease outbreaks across the continent, where diagnostic laboratories must scale up testing rapidly while maintaining rigorous biosafety standards, often without corresponding increases in resources, staffing or infrastructure (10).

In Somalia, the health system is recovering from more than three decades of conflict, state fragility and underinvestment, and laboratory services are central to disease diagnosis, clinical decision-making and outbreak detection. Biosafety standards and laboratory quality management systems vary widely across institutions, reflecting the broader fragmentation of health service delivery in the post-conflict Somali health sector. The available Somali evidence is narrow in scope. The most directly relevant assessment applied the WHO Infection Prevention and Control Assessment Framework to Somali health facilities and documented substantial gaps in biosafety infrastructure, guideline availability and programme governance (11). That work is facility-level and structural: it characterizes what institutions have in place. It does not measure what individual laboratory professionals know, what they report doing at the bench, or whether the two correspond. Three gaps therefore remain. First, individual-level biosafety knowledge among Somali laboratory professionals has not been quantified with a structured, scored instrument. Second, self-reported biosafety practice has not been measured alongside knowledge in the same participants, so the knowledge–practice relationship is unknown. Third, no published study has recruited from both public and private hospitals within a single sampling frame.

The public–private distinction is substantively important in this setting. The two sectors differ in financing model, staffing establishment and turnover, procurement reliability for PPE and safety consumables, exposure to external regulatory inspection, and the presence of formal quality management structures. Hospital ownership is therefore a plausible structural determinant of biosafety practice, and one actionable through regulation and licensing. Because much laboratory service delivery in Mogadishu occurs in private facilities, evidence drawn exclusively from public hospitals would describe a minority of the workforce.

Accordingly, this study aimed to: (i) assess laboratory biosafety knowledge; (ii) assess self-reported laboratory biosafety practice; (iii) examine the association between knowledge and self-reported practice; and (iv) identify individual, professional and institutional factors associated with good self-reported practice among medical laboratory professionals working in selected public and private hospitals in Mogadishu, Somalia.

## Methods

### Study design and setting

A facility-based cross-sectional study was conducted among medical laboratory professionals working in selected hospitals in Mogadishu, Somalia. Mogadishu hosts a small number of public hospitals and several private hospitals providing routine diagnostic, emergency and reference laboratory services; these facilities vary considerably in size and capacity. At the time of the study, the sampling frame of hospitals operating a functional clinical laboratory and employing at least two laboratory professionals comprised 45 facilities (11 public and 34 private), compiled from ministry of health (12). Data collection was carried out between September 2025 and October 2025 across 12 hospitals selected as described below.

### Study population and eligibility criteria

The study population comprised all medical laboratory professionals employed in the selected hospitals during the study period. Eligible participants were laboratory technicians, technologists, laboratory scientists and supervisors who had worked in their respective facilities for at least 3 months. Laboratory personnel on annual, maternity or sick leave, as well as interns and students, were excluded.

### Sampling procedure

A two-stage approach was used. In the first stage, 12 hospitals were selected from the sampling frame by stratified simple random sampling, with stratification by ownership so that both sectors were represented; 4 of 11 eligible public hospitals and 8 of 34 eligible private hospitals were drawn using a computer-generated random number sequence. In the second stage, all eligible medical laboratory professionals employed in the selected hospitals (*N* = 164) were invited to participate; no further sampling was undertaken at this level. Updated staff lists were obtained from each hospital laboratory manager to enumerate all professionals meeting the inclusion criteria.

This design is a complete enumeration of eligible professionals within the sampled hospitals, not a census of all medical laboratory professionals in Mogadishu. Complete enumeration at the second stage removes sampling error within participating facilities and maximises the available sample size, but representativeness of the wider workforce depends on the first-stage selection of hospitals. The number of professionals contributed by each participating hospital ranged from 5 to 26. Because participants are clustered within hospitals, observations within a facility may be more similar to one another than to those from other facilities; this is addressed in the analysis and limitations sections.

### Data collection instrument and validation

Data were collected using a structured, self-administered questionnaire developed from the WHO Laboratory Biosafety Manual (1) and previously validated knowledge-and-practice instruments from comparable studies (13–15). The questionnaire comprised three sections: (1) socio-demographic and professional characteristics; (2) knowledge of laboratory biosafety, assessed with 8 single-best-answer multiple-choice items; and (3) laboratory biosafety practice, assessed with 12 dichotomous Yes/No items, of which 9 contribute to the composite practice score (see below).

Prior to the main data collection, the questionnaire was pre-tested on 16 laboratory professionals (approximately 10% of the target population) in a comparable hospital not included in the study. Feedback was used to refine wording, improve clarity and ensure contextual relevance. Pre-testing established comprehensibility and feasibility and is not equivalent to psychometric validation; internal consistency was therefore assessed in the full study sample. Because both the knowledge and the practice items are dichotomously scored, the Kuder–Richardson Formula 20 (KR-20) coefficient was computed for each scale; for dichotomous items KR-20 is mathematically identical to Cronbach’s alpha. KR-20 was 0.60 for the 8-item knowledge scale and 0.52 for the 9-item practice scale. Both fall below the conventional 0.70 threshold, and this is addressed explicitly in the Limitations. The full questionnaire and the complete scoring key are available from the corresponding author on reasonable request. Questionnaires were administered during working hours and retrieved on the same day or within 1–2 days to minimise non-response and were reviewed for completeness before data entry.

### Variables and measurement

#### Knowledge of laboratory biosafety

Knowledge was assessed with 8 items covering eight domains: common laboratory-acquired infections; the purpose of personal protective equipment; unsafe practices in handling biological specimens; appropriate use of a biosafety cabinet; color coding for infectious waste containers; laboratory handwashing practice; response to chemical spills; and the institutions responsible for developing and enforcing laboratory safety guidelines. Each item was a single-best-answer multiple-choice question with exactly one correct response; no item permitted multiple correct selections and no item was partially credited. Each correct answer was scored 1 and each incorrect answer 0, giving a total score from 0 to 8.

Total knowledge scores were classified using the modified Bloom’s cut-off points, widely applied in knowledge–attitude–practice research in comparable settings (8, 13, 15): good knowledge, ≥80% of the maximum (≥6.4, i.e., 7–8 correct); moderate knowledge, 60–79.9% (4.8–6.39, i.e., 5–6 correct); and poor knowledge, <60% (<4.8, i.e., ≤4 correct). These thresholds were fixed *a priori* and are not derived from the sample distribution.

#### Self-reported practice of laboratory biosafety

Practice was assessed with 12 dichotomous Yes/No items. Three items were excluded from the composite score. Receipt of formal biosafety training and participation in a safety drill in the preceding year are institutional exposures rather than individual safety behaviors and including them would conflate determinants of practice with practice itself; both are instead analyzed as independent variables. A third item, self-rated knowledge of what to do in the event of fire or chemical spill, measures perceived competence rather than enacted behavior and was likewise excluded. The composite practice score therefore comprises 9 items, each scored 1 for the safe response and 0 otherwise, giving a range of 0 to 9, with higher scores indicating better self-reported adherence. One item eating or drinking inside the laboratory is negatively worded and was reverse-scored, so that a higher contribution always denotes safer practice. In Table 1 this item is reported after reverse-coding for consistency.

**Table 1:** Self-reported laboratory biosafety practice among medical laboratory professionals (*N* = 164).

Practice item	Safe practice, *n* (%)^a^	Item level^b^
Wearing a laboratory coat while working	161 (98.2)	Good
Using gloves while handling specimens or reagents	160 (97.6)	Good
Refraining from eating or drinking in the laboratory (reverse-scored)	146 (89.0)	Good
Disposing of sharps in puncture-resistant containers	145 (88.4)	Good
Washing hands before and after laboratory procedures	161 (98.2)	Good
Reporting laboratory accidents (e.g., spills, needle-stick injuries)	134 (81.7)	Good
Following standard operating procedures (SOPs)	158 (96.3)	Good
Regularly inspecting safety equipment	129 (78.7)	Moderate
Keeping the laboratory workstation clean and organised	160 (97.6)	Good
Reported separately—not included in the composite practice score:		
Received formal biosafety training (analysed as an independent variable)	154 (93.9)	—
Participated in a safety drill in the past year (independent variable)	106 (64.6)	—
Knows what to do in case of fire or chemical spill (perceived competence)	143 (87.2)	—
Total practice score (range 0–9)^c^	Median 9 (IQR 8–9)	**—**

a Practice items were scored 1 = safe response and 0 = otherwise and are reported as frequencies and percentages. The negatively worded item on eating or drinking inside the laboratory was reverse-scored and is presented after reverse-coding, so that for every item a higher proportion denotes safer practice; 18 participants (11.0%) reported eating or drinking in the laboratory. The composite practice score is the sum of the nine items above the divider (range 0–9).

b Item-level classification applies the modified Bloom’s cut-off points defined in the Methods: good, ≥80%; moderate, 60–79.9%; poor, <60%. Classification is deliberately not applied to the three items below the divider, and these cells are marked with a dash rather than left blank: receipt of formal training and participation in a safety drill measure institutional exposure, and knowledge of what to do in a fire or chemical spill measures perceived competence, whereas the cut-off points classify enacted practice and are not interpretable for these constructs. The proportions for all three items are nevertheless reported in full above.

c The final row summarises the total practice score across participants. Because the total score departed significantly from normality (Shapiro–Wilk *W* = 0.703, *p* < 0.001), central tendency is reported as the median with interquartile range; the mean and standard deviation are not reported.

Composite practice scores were classified using the same modified Bloom’s cut-off points applied to the knowledge scale, so that the two constructs are categorized on a common percentage-of-maximum basis: good practice, ≥80% of the maximum (≥7.2, i.e., 8–9 items); moderate practice, 60–79.9% (5.4–7.19, i.e., 6–7 items); and poor practice, <60% (<5.4, i.e., ≤5 items).

Item-level and participant-level classifications are distinct and are labelled separately throughout. In Tables 1, 2 the “level” column classifies the performance of the group on a single item—the percentage of respondents answering correctly or reporting the safe practice. In Table 3 the classification applies to each individual participant’s total score. The two are not interchangeable, and item-level labels do not describe any individual respondent.

**Table 2:** Knowledge of laboratory biosafety among laboratory professionals in Mogadishu hospitals (*N* = 164).

Knowledge item	Correct, *n* (%)^a^	Incorrect, *n* (%)	Item level^b^
Common laboratory-acquired infections	131 (79.9)	33 (20.1)	Moderate
Primary purpose of personal protective equipment	142 (86.6)	22 (13.4)	Good
Unsafe practices when handling biological specimens	128 (78.0)	36 (22.0)	Moderate
Appropriate use of a biosafety cabinet	122 (74.4)	42 (25.6)	Moderate
Colour coding for infectious waste containers	88 (53.7)	76 (46.3)	Poor
Proper handwashing practice in the laboratory	146 (89.0)	18 (11.0)	Good
Appropriate response to chemical spills	130 (79.3)	34 (20.7)	Moderate
Institutions developing and enforcing safety guidelines	118 (72.0)	46 (28.0)	Moderate
Total knowledge score (range 0–8)^c^	Median 7 (IQR 5–7)	**—**	**—**

a Items were scored 1 = correct and 0 = incorrect and are reported as frequencies and percentages.

b Item-level classification describes the performance of the sample on a single item and applies the modified Bloom’s cut-off points: good, ≥80% correct; moderate, 60–79.9%; poor, <60%. Item-level classification is distinct from the participant-level classification of total scores used in Table 3 and does not describe any individual respondent.

c The final row summarises the total knowledge score across participants. Because the total score departed significantly from normality (Shapiro–Wilk *W* = 0.888, *p* < 0.001), central tendency is reported as the median with interquartile range; the mean and standard deviation are not reported.

**Table 3:** Association between knowledge level and self-reported practice level (*N* = 164).

Knowledge level^a^	Good practice, *n* (%)	Sub-optimal practice, *n* (%)^b^	Total, *n* (%)
Good (7–8 correct)	78 (94.0)	5 (6.0)	83 (50.6)
Moderate (5–6 correct)	38 (74.5)	13 (25.5)	51 (31.1)
Poor (≤4 correct)	18 (60.0)	12 (40.0)	30 (18.3)
Total	134 (81.7)	30 (18.3)	164 (100.0)

*χ*^2^ (df = 2) = 19.584; *p* < 0.001; Cramer’s *V* = 0.346; minimum expected cell count = 5.49. Spearman’s *ρ* between the untransformed knowledge and practice scores = 0.296, *p* < 0.001.

a Participant-level classification of total knowledge score (range 0–8) using modified Bloom’s cut-off points.

b Practice categories were collapsed from three levels into good versus sub-optimal because the full 3 × 3 cross-tabulation produced expected cell counts below 5.

#### Independent variables

Independent variables comprised age group, sex, educational qualification, years of professional experience, job category, hospital ownership (public versus private), receipt of formal biosafety training, participation in a safety drill in the preceding year, and the total knowledge score. Hospital ownership was assigned from the facility name using the classification recorded on the study questionnaire.

### Data management and analysis

Completed questionnaires were coded and exported to IBM SPSS Statistics version 20 for analysis. Data were double-entered and discrepancies resolved against the source questionnaires. All 164 returned questionnaires were complete on the knowledge and practice items, so no imputation was required and all knowledge and practice analyses use the full denominator of 164. Job category was missing for two participants, and analyses involving that variable use a denominator of 162; the denominator for every analysis is stated in the corresponding table.

The distribution of the total knowledge and practice scores was examined using the Shapiro Wilk test, supplemented by histograms and normal Q–Q plots. Both scores departed significantly from normality (knowledge *W* = 0.888, *p* < 0.001; practice *W* = 0.703, *p* < 0.001), so continuous scores are summarized as medians with interquartile ranges and non-parametric methods are used throughout. Categorical variables are summarized as frequencies and percentages. Because both total scores departed significantly from normality, means and standard deviations are not reported for the knowledge or practice scores at either the item or the total level, and all measures of central tendency are reported as medians with interquartile ranges. Dichotomously scored items are summarized as frequencies and percentages alone: under 0/1 coding the item mean is numerically identical to the proportion giving the correct or safe response, so reporting it alongside that proportion would duplicate the same quantity in a form that invites comparison with the total score on a different scale. Binary logistic regression was retained despite the non-normal score distributions, as it makes no distributional assumption about its predictor variables.

The association between overall knowledge level and overall practice level was examined using the Pearson chi-square test, with significance defined at *p* < 0.05, and strength of association assessed with Cramer’s *V*. Chi-square assumptions, including minimum expected cell counts, were verified before interpretation. Because categorizing continuous scores discards information and reduces power, the relationship between the knowledge and practice scores was additionally examined in untransformed form using Spearman’s rank correlation. The correlational and categorical analyses are reported as complementary rather than alternative analyses.

Factors associated with good self-reported practice were examined using binary logistic regression, with practice dichotomised as good versus sub-optimal (moderate and poor combined). Candidate variables were age group, sex, educational qualification, years of professional experience, job category, hospital ownership, receipt of formal biosafety training, participation in a safety drill, and the total knowledge score. Each was first examined in univariable analysis; variables associated with the outcome at *p* < 0.25 were entered into a multivariable model. Multicollinearity was assessed using variance inflation factors (threshold >5; all values were ≤1.07) and model calibration using the Hosmer–Lemeshow goodness-of-fit test. Results are reported as crude and adjusted odds ratios with 95% confidence intervals. Because participants were clustered within 12 hospitals, and the number of clusters is too small to support reliable estimation of cluster-robust standard errors, clustering was not accounted for in the models; this is acknowledged as a limitation.

### Ethical considerations

Ethical approval was obtained from the National Institute of Health, Somalia (Approval No. NIH/IRB/96/AUG/2025). Written informed consent was obtained from all participants prior to data collection. Confidentiality and anonymity were strictly maintained throughout.

## Results

### Sociodemographic and professional characteristics

A total of 164 medical laboratory professionals participated, corresponding to a response rate of 100% of those enumerated in the participating hospitals (Table 4). Most respondents were young adults, with 48.8% aged 26–35 years and 45.1% aged 18–25 years. The sex distribution was nearly equal (51.2% male; 48.8% female). Most participants held a bachelor’s degree (77.4%), while 22.6% held a postgraduate degree. Laboratory technicians constituted the largest professional group (70.4% of the 162 participants with this item recorded), followed by laboratory supervisors (15.4%). Over half of respondents (56.7%) had 1–5 years of professional experience and 24.4% had 6–10 years. Ninety-nine participants (60.4%) were employed in private hospitals and 65 (39.6%) in public hospitals. The number of participants contributed by each hospital ranged from 5 to 26.

**Table 4:** Sociodemographic and professional characteristics of study participants (*N* = 164).

Variable	Category	Frequency (*n*)	Percentage (%)
Age (years)	18–25	74	45.1
	26–35	80	48.8
	36–45	8	4.9
	46 and above	2	1.2
Sex	Male	84	51.2
	Female	80	48.8
Educational qualification	Bachelor’s degree	127	77.4
	Postgraduate degree	37	22.6
Role in medical laboratory^a^	Laboratory supervisor	25	15.4
	Laboratory technician	114	70.4
	Laboratory assistant	4	2.5
	Trainer/volunteer	19	11.7
Work experience (years)	<1	13	7.9
	1–5	93	56.7
	6–10	40	24.4
	>10	18	11.0
Hospital ownership	Public hospital	65	39.6
	Private hospital	99	60.4

a Role was not recorded for two participants; percentages for this variable use a denominator of 162. Hospital ownership was assigned from the facility name using the public/private classification recorded on the study questionnaire.

### Overall knowledge and practice scores

Total knowledge scores ranged from 1 to 8 of a possible 8, with a median of 7 (IQR 5–7); the Shapiro–Wilk test did not support the assumption of normality (*W* = 0.888, *p* < 0.001), so the median and interquartile range are reported as the measure of central tendency and no mean or standard deviation is given. Applying the pre-specified thresholds, 83 participants (50.6%) were classified as having good knowledge, 51 (31.1%) moderate knowledge and 30 (18.3%) poor knowledge.

Total practice scores ranged from 4 to 9 of a possible 9, with a median of 9 (IQR 8–9); this score likewise departed significantly from normality (Shapiro–Wilk W = 0.703, *p* < 0.001), and is therefore also summarised as the median with interquartile range. Ninety-two participants (56.1%) achieved the maximum score of 9. Applying the same thresholds, 134 participants (81.7%) were classified as having good practice, 24 (14.6%) moderate practice and 6 (3.7%) poor practice. All knowledge and practice analyses use a denominator of 164.

### Knowledge of laboratory biosafety

Overall, laboratory professionals demonstrated a moderate level of biosafety knowledge (Table 2). High levels of knowledge were observed for handwashing practice (89.0%) and for the purpose of personal protective equipment (86.6%), reflecting good awareness of essential safety measures. Moderate knowledge was observed for laboratory-acquired infections, unsafe specimen handling, chemical spill response, biosafety cabinet use and the institutions responsible for safety guidelines, with correct responses ranging from 72.0 to 79.9%. Knowledge of infectious waste color-coding was comparatively low (53.7%), indicating a critical gap in waste management knowledge: 55 participants (33.5%) selected red rather than yellow.

### Self-reported practice of laboratory biosafety

Self-reported practice was high across most items (Table 1). Reported compliance exceeded 95% for wearing a laboratory coat (98.2%), washing hands before and after procedures (98.2%), using gloves (97.6%), keeping the workstation clean (97.6%) and following standard operating procedures (96.3%). Lower proportions reported refraining from eating or drinking in the laboratory (89.0%), disposing of sharps in a puncture-resistant container (88.4%), reporting laboratory accidents (81.7%) and regularly inspecting safety equipment (78.7%), the last falling into the moderate band.

Of the three items reported separately because they are exposures or perceived competence rather than enacted behaviors, 154 participants (93.9%) reported having received formal biosafety training and 143 (87.2%) reported knowing what to do in the event of fire or chemical spill, but only 106 (64.6%) had participated in a safety drill in the preceding year—the lowest figure recorded on any item in the instrument.

### Association between knowledge and self-reported practice

For the association analysis the three practice categories collapsed into good versus sub-optimal practice (moderate and poor combined), because the full 3 × 3 cross-tabulation produced expected cell counts below 5. The collapsed 3 × 2 table satisfies the chi-square assumption, with a minimum expected count of 5.49, and the categories are labelled ‘good’ and ‘sub-optimal’ in Table 3 to make the collapsing explicit. The three-level practice distribution is reported in full above.

A clear gradient was observed across knowledge levels. Among participants with good knowledge, 94.0% reported good practice; among those with moderate knowledge, 74.5%; and among those with poor knowledge, 60.0% (Table 3). Pearson’s chi-square analysis showed a statistically significant association between knowledge level and self-reported practice level (*χ*^2^ = 19.58, df = 2, *p* < 0.001), and the strength of association was moderate (Cramer’s *V* = 0.35). The complementary analysis using untransformed scores gave a significant positive Spearman rank correlation between total knowledge and total practice scores (*ρ* = 0.296, *p* < 0.001), consistent with the categorical result.

### Factors associated with good self-reported practice

Table 5 presents the univariable and multivariable logistic regression analyses. In univariable analysis, the total knowledge score was strongly associated with good practice (cOR 1.61 per additional correct answer, 95% CI 1.27–2.04, *p* < 0.001). Participation in a safety drill (cOR 2.12, 95% CI 0.95–4.72, *p* = 0.067), receipt of formal training (cOR 3.28, 95% CI 0.86–12.45, *p* = 0.081) and job category (technician versus other roles, cOR 0.51, 95% CI 0.19–1.34, *p* = 0.173) met the *p* < 0.25 threshold and were carried forward. Job category was entered as technician versus all other roles rather than as four separate categories, because every laboratory supervisor in the sample reported good practice, producing complete separation and non-convergence when the four-category form was modeled. Age, sex, educational qualification, years of experience and hospital ownership were not associated with the outcome and were not entered into the multivariable model.

**Table 5:** Factors associated with good self-reported laboratory biosafety practice: univariable and multivariable binary logistic regression.

Variable	Crude OR (95% CI)	*p*	Adjusted OR (95% CI)	*p*
Total knowledge score (per 1-point increase)	1.61 (1.27–2.04)	<0.001	1.73 (1.33–2.24)	<0.001
Participated in a safety drill (yes vs. no)	2.12 (0.95–4.72)	0.067	2.80 (1.09–7.15)	0.032
Received formal biosafety training (yes vs. no)	3.28 (0.86–12.45)	0.081	2.13 (0.43–10.47)	0.352
Job category (technician vs. other roles)	0.51 (0.19–1.34)	0.173	0.55 (0.19–1.57)	0.265
Sex (female vs. male)	1.55 (0.69–3.46)	0.289	Not entered	—
Hospital ownership (private vs. public)	1.21 (0.54–2.69)	0.647	Not entered	—
Educational qualification (postgraduate vs. bachelor’s)	0.76 (0.31–1.88)	0.553	Not entered	—
Age group (≥26 vs. 18–25 years)	1.08 (0.49–2.39)	0.851	Not entered	—
Work experience (>5 vs. ≤ 5 years)	0.93 (0.41–2.12)	0.869	Not entered	—

OR, odds ratio; CI, confidence interval. Outcome: good practice (total score 8–9 of 9) versus sub-optimal practice (≤7). Univariable models use *N* = 164 except job category (*n* = 162). Variables associated with the outcome at *p* < 0.25 in univariable analysis were entered into the multivariable model (*n* = 162). Hosmer–Lemeshow *χ*^2^ = 5.28, df = 8, *p* = 0.728; Nagelkerke *R*^2^ = 0.236; all variance inflation factors ≤1.07. Job category was modeled as technician versus all other roles; the four-category form produced complete separation because no laboratory supervisor reported sub-optimal practice.

In the multivariable model (*n* = 162), two variables remained independently associated with good self-reported practice. Each additional correct knowledge answer increased the odds of good practice by 73% (aOR 1.73, 95% CI 1.33–2.24, *p* < 0.001), and participants who had taken part in a safety drill in the preceding year had almost three times the odds of good practice compared with those who had not (aOR 2.80, 95% CI 1.09–7.15, *p* = 0.032). Receipt of formal training was no longer associated with the outcome once drill participation was accounted for (aOR 2.13, 95% CI 0.43–10.47, *p* = 0.352), nor was job category (aOR 0.55, 95% CI 0.19–1.57, *p* = 0.265). The model was well calibrated (Hosmer–Lemeshow *χ*^2^ = 5.28, df = 8, *p* = 0.728) and explained a modest proportion of variance (Nagelkerke *R*^2^ = 0.236). All variance inflation factors were ≤1.07, indicating no problematic multicollinearity.

## Discussion

This study provides one of the first assessments of laboratory biosafety knowledge and self-reported practice among medical laboratory professionals working in both public and private hospitals in Mogadishu, Somalia. The findings reveal moderate biosafety knowledge alongside high self-reported practice, a statistically significant positive association between the two, and an independent association between recent safety drill participation and good practice.

Respondents demonstrated relatively strong knowledge of fundamental biosafety principles, including hand hygiene, appropriate use of personal protective equipment and awareness of laboratory-acquired infections. These findings are consistent with reports from other low- and middle-income countries, where laboratory professionals often possess theoretical awareness of basic biosafety concepts as a result of pre-service education or informal workplace exposure (13). Similar assessments of safety requirements and actual laboratory practices have been documented in health institute teaching laboratories in comparable settings (16). The relatively high awareness of PPE and hand hygiene may reflect the global emphasis on infection prevention during recent infectious disease outbreaks, including COVID-19 (9, 17).

Notable deficiencies were nonetheless observed in participants’ understanding of infectious waste segregation, particularly the correct application of color-coding systems. Just over half answered this item correctly, and a third selected red rather than yellow. This aligns with evidence from sub-Saharan Africa showing that inadequate training in biomedical waste management contributes to suboptimal practice and poor compliance with biosafety standards among healthcare workers (18).

A study conducted in Ethiopia found that limited availability of color-coded bins and gaps in training were significantly associated with lower levels of correct waste segregation practice (19). Inadequate segregation of infectious healthcare waste elevates the risk of occupational exposure and environmental contamination, with documented impacts on human health and the environment (20), particularly in low-resource health systems where waste treatment and final disposal capacity is insufficient or poorly regulated (11).

In contrast to much of the published knowledge–practice literature, this study found a statistically significant positive association between categorized biosafety knowledge and self-reported practice, with a clear monotonic gradient: 94.0% of those with good knowledge, 74.5% of those with moderate knowledge and 60.0% of those with poor knowledge reported good practice. The association was confirmed on the untransformed scores (*ρ* = 0.296, *p* < 0.001) and persisted in the multivariable model, where each additional correct knowledge answer raised the odds of good practice by 73%. The strength of association was moderate rather than strong (Cramer’s *V* = 0.35; Nagelkerke *R*^2^ = 0.237), meaning that knowledge accounted for part but by no means all of the variation in reported practice. This finding requires careful interpretation, and in particular it should not be read as evidence that knowledge transfer alone will secure safe practice. Three considerations temper the result. First, both constructs were measured by the same self-administered instrument at the same sitting, so shared method variance and consistency motivation may inflate the observed association. Second, the practice measure exhibits a pronounced ceiling effect, with 56.1% of participants achieving the maximum score and 81.7% classified as having good practice; restriction of range at the upper end of the scale limits what the association can reveal about the determinants of poor practice specifically. Third, an association observed in cross-sectional data cannot establish direction: greater knowledge may promote safer practice, but working in a laboratory with strong safety routines may equally promote the acquisition of knowledge.

The wider occupational health literature remains relevant here. Systematic reviews have concluded that knowledge of correct infection prevention and control procedures is frequently dissociated from actual compliance, with organisational support, resource availability and workplace culture playing a decisive role in shaping practice (5). Evidence from South Asian healthcare settings demonstrates that laboratory safety protocols and quality management systems are essential components for translating knowledge into practice (21). Similar dissociations have been documented among nursing personnel, in whom high scores on knowledge of precautionary measures have coexisted with markedly lower observed adherence (6, 9), and studies from Ethiopia and other African contexts report weak or absent correlations between biosafety knowledge and compliance (14). Studies from South Asian healthcare settings similarly show knowledge-practice gaps in infection prevention and control among healthcare personnel (22). The present finding of a moderate positive association does not contradict this body of work so much as qualify it: knowledge appears to matter in this workforce but explains under a quarter of the variance in the outcome, leaving the greater part attributable to factors not captured by an individual knowledge test.

The independent association between participation in a safety drill in the preceding year and good self-reported practice is, in policy terms, the most actionable finding of this study. Drill participation nearly tripled the odds of good practice after adjustment (aOR 2.80, 95% CI 1.09–7.15), and it was the lowest-scoring item in the entire instrument: only 64.6% of participants had taken part in a drill in the past year, against 93.9% who reported having received formal biosafety training at some point. Notably, receipt of training lost its univariable association with practice once drill participation was included in the model, which suggests that recent, active, institutionally organised safety activity is more closely tied to reported behavior than the historical fact of having been trained. This is consistent with WHO observations that biosafety training in resource-constrained settings is often irregular, donor-dependent or one-off (1), and with reports that health workforce preparedness in fragile settings is constrained less by awareness than by the absence of sustained in-service systems (7).

It is important to distinguish clearly between what these data demonstrate and what is inferred from the wider literature. This study demonstrates the levels of knowledge and self-reported practice reported above, the positive association between them, and the independent associations of knowledge score and drill participation with good practice. Explanations invoking prolonged health system fragility, inconsistent regulatory oversight, the absence of standardised biosafety management systems and limited accountability mechanisms are plausible and are supported by facility-level assessments of the Somali health system (14) and by the wider literature (6, 10), but they were not themselves tested here and are advanced as hypotheses for future work. Hospital ownership, which was tested, showed no association with practice (cOR 1.21, 95% CI 0.54–2.69), which is itself informative: the structural differences between the public and private sectors did not translate into detectable differences in reported biosafety behavior in this sample.

Because self-reported practice is the principal outcome of this study, the limitations of self-report deserve particular emphasis rather than passing acknowledgement. Participants were asked to report behaviors that are explicitly normative within their profession, in a questionnaire administered at their place of work; under these conditions socially desirable responding is a substantial and predictable threat to validity. Its likely direction is upward, and the extreme ceiling observed here 98.2% reporting always wearing a laboratory coat and always washing hands, 56.1% reporting the maximum score on every scored item—is more plausibly a signature of that bias than an accurate picture of bench practice in facilities that the WHO assessment framework found to have substantial infrastructural gaps (14). Studies comparing self-reported with directly observed infection-control behavior have consistently found self-report to overestimate compliance (6, 8). The deficits identified here should therefore be regarded as conservative estimates of the true deficits, and the reported practice levels should not be read as a measurement of observed laboratory compliance. Anonymity was assured and no identifying information was collected, which mitigates but does not eliminate this bias; no social desirability scale was administered, so its magnitude cannot be quantified in these data. The title, abstract and conclusions have been framed accordingly.

This study has several limitations. First, the cross-sectional design precludes causal inference regarding the relationship between knowledge, institutional factors and safety practice, and cannot establish the direction of the observed knowledge–practice association. Second, both knowledge and practice were assessed using a self-administered, self-reported instrument, subject to social desirability bias as discussed above; direct observational verification through structured workplace audits was not performed. Third, the practice scale shows a marked ceiling effect that restricts variance in the outcome and limits the power of the regression to detect determinants of poor practice. Fourth, the internal consistency of both scales was below the conventional 0.70 threshold (KR-20 = 0.60 for knowledge and 0.52 for practice); short dichotomous scales characteristically yield lower coefficients, but the practice scale in particular should be regarded as a set of related indicators rather than a psychometrically robust unidimensional measure, and the composite scores should be interpreted with corresponding caution. Fifth, participants were recruited from 12 hospitals in Mogadishu only, so the findings describe eligible professionals in the sampled facilities and may not generalise to the wider Mogadishu workforce, still less to other regions of Somalia. Sixth, participants are clustered within hospitals, and facility-level factors such as procurement reliability and supervisory practice are likely to induce within-hospital correlation; with only 12 clusters this could not be reliably modeled, so standard errors may be understated and the confidence intervals reported here are correspondingly optimistic. Finally, several constructs implicated in the discussion safety culture, supervisory intensity, workload, PPE availability and procurement continuity were not measured, and their role in shaping practice in this setting remains untested.

## Conclusion

Medical laboratory professionals in Mogadishu hospitals demonstrated moderate biosafety knowledge and high self-reported biosafety practice, although there were gaps in knowledge of infectious waste color-coding. Knowledge scores were positively associated with self-reported practice, while recent participation in a safety drill was independently associated with reported participants’ practice. These findings suggest that regular safety drills and targeted reinforcement of infectious waste-management procedures may be useful areas for strengthening biosafety practices. However, the high reported practice scores and their potential effect underscore the need for future studies to assess biosafety practices through direct observation.

## Data Availability

The raw data supporting the conclusions of this article will be made available by the authors, without undue reservation.

## References

[1] World Health Organization. (2020). Laboratory Biosafety Manual (4th). World Health Organization. Available online at: https://www.who.int/publications/i/item/9789241011433 (Accessed January 15, 2022).

[2] Pottage T Glendinning S. The biosafety level requirement in biological research laboratories: focus on procedures for handling contaminated sharps and related laboratory-acquired infection risks. J Hosp Infect. (2022) 122:126–32. doi: 10.1016/j.jhin.2022.01.005, 35041896

[3] Bukasa SK Mwanza A Mukendi PK Heger S Lepowitch JI Pierce KE . Risk factors and mitigation strategies of laboratory-acquired infections in research and clinical laboratories worldwide: a systematic review. Lancet Microbe. (2025) 6:101–12. 10.1016/j.lanmic.2025.101157 40609561

[4] Sieber R Grube M Zeller S. Laboratory-acquired infections in clinical practice: epidemiology, risk assessment and management strategies. Swiss Med Wkly. (2023) 153:w30004. doi: 10.4414/smw.2023.w30004

[5] Houghton C Meskell P Delaney H Murphy K O'Neill H Webster J. Knowledge of infection prevention and control among healthcare workers and factors influencing compliance: a systematic review. Antimicrob Resist Infect Control. (2021) 10:86. doi: 10.1186/s13756-021-00957-0, 34082822 PMC8173512

[6] Kulkarni PA Greene MT Saint S Fowler KE Jacob S Dillon L . Comparing observed with self-reported hand hygiene adherence among bedside nurses and physicians at two hospitals in the United States. Am J Infect Control. (2026) 54:241–6. doi: 10.1016/j.ajic.2025.10.032, 41207631 PMC13051545

[7] Okoroafor SC Asamani JA Kabego L Ahmat A Nyoni J Millogo JJS . Preparing the health workforce for future public health emergencies in Africa. BMJ Glob Health. (2022) 7:e008327. doi: 10.1136/bmjgh-2021-008327, 35414522 PMC9006823

[8] Lake EA Demissie BW Gebeyehu NA Wassie AY Gelaw KA Azeze GA. Knowledge, attitude and practice towards COVID-19 among health professionals in Ethiopia: a systematic review and meta-analysis. PLoS One. (2021) 16:e0247204. doi: 10.1371/journal.pone.0247204, 33606744 PMC7894858

[9] Chittim B Murakami T Nnamdi N Obi LU Adeyiga JE Owolabi SM . Strengthening biosafety training and resource availability in sub-Saharan African laboratories: challenges and opportunities. Infect Control Hosp Epidemiol. (2024) 45:832–9. doi: 10.1086/720815

[10] Karamitsos T Pillay A van Rensburg EJ. Workforce preparedness and pandemic response capacity in African diagnostic laboratories: a systematic review. Lancet Microbe. (2024) 5:e145–54. doi: 10.1016/S2666-5247(23)00326-7

[11] Adam AA Mohamed HD Anyiam FE. Assessment of infection prevention and control in Somali healthcare facilities using the WHO infection prevention and control assessment framework. Antimicrob Resist Infect Control. (2025) 14:103. doi: 10.1186/s13756-025-01584-9, 40898374 PMC12403908

[12] National Report. Somalia Harmonized Health Facility Assessment 2022–2023. (2023). Available online at: https://data-archive.hhfa.online/index.php/catalog/144/related-materials (accessed August 31, 2026)

[13] Islam MN Kabir MH Yusuf MA Salam MB Perveen I Shirin S. Knowledge of biosafety measures among laboratory personnel at tertiary level public hospitals in Dhaka City. Bangladesh J Infect Dis. (2020) 7:49–56. doi: 10.3329/bjid.v7i2.51509

[14] Tadesse MT Ayalke LL Tedla BF Weldemeskel FA Yirdaw AS Abebe TA. Biosafety management practices among clinical laboratory professionals in Debre Berhan town governmental hospitals, Ethiopia: a cross-sectional study. Health Sci Rep. (2025) 8:e70361. doi: 10.1002/hsr2.70361, 39872910 PMC11770472

[15] Torp KM Lesmeister K Vega CJ. Health-care-associated infections and occupational safety measures in clinical laboratories. Healthcare (Basel). (2024) 11:242. doi: 10.3390/healthcare11020242

[16] Aliyo A Edin A. Assessment of safety requirements and their practices among teaching laboratories of health institutes. Microbiol Insights. (2023) 16:11786361231174414. doi: 10.1177/11786361231174414, 37205985 PMC10189843

[17] Okafor NI Ihim SA Menkiti AN. Waste management practices in healthcare facilities: a review of challenges and solutions in developing countries. Univ J Manag Res. (2025) 5:35–48.

[18] Ibrahim Y Nneka AK Lateef F Shuaibu MA Samuel AA. Gender disparities and practice gaps in healthcare waste management among Gombe state health workers. UMYU J Microbiol Res. (2025) 10:563–71. doi: 10.47430/ujmr.25103.053

[19] Mitiku G Admasie A Birara A Yalew W. Biomedical waste management practices and associated factors among healthcare workers in the era of the COVID-19 pandemic at metropolitan city private hospitals, Amhara region, Ethiopia, 2020. PLoS One. (2020) 17:e0266037. doi: 10.1371/journal.pone.0266037, 35385508 PMC8985930

[20] Addo KK Mensah-Attipoe IK Bamford-Addo SA. Biomedical waste management practices and its impact on human health and the environment: a review. J Environ Health. (2020) 82:14–21. doi: 10.1002/fsn3.70575

[21] Garcia-Martinez JL Smith R Johnson K. Laboratory safety protocols and quality management systems: evidence from south Asian healthcare settings. BMC Health Serv Res. (2019) 4:316. doi: 10.1186/s13104-019-4316-y

[22] Kiddeer M Abbas A Basit A Ahmad T Masood I. Assessment of knowledge, attitude and practices regarding standard precautions of infection prevention and control and associated factors among healthcare personnel working at tertiary care hospitals in Punjab, Pakistan. Infect Prev Pract. (2025) 7:100464. doi: 10.1016/j.infpip.2025.100464, 40636400 PMC12240071

